# Characterization of Choline Nutriture among Adults and Children with Phenylketonuria

**DOI:** 10.3390/nu14194056

**Published:** 2022-09-29

**Authors:** Meriah S. Schoen, Usha Ramakrishnan, Jessica A. Alvarez, Thomas R. Ziegler, Xiangqin Cui, Rani H. Singh

**Affiliations:** 1 Nutrition and Health Sciences, Laney Graduate School, Emory University, Atlanta, GA 30322, USA; 2 Department of Human Genetics, Emory University, Atlanta, GA 30322, USA; 3 Hubert Department of Global Health, Rollins School of Public Health, Emory University, Atlanta, GA 30322, USA; 4 Department of Medicine, Emory University, Atlanta, GA 30322, USA; 5 Biostatistics and Bioinformatics, Rollins School of Public Health, Emory University, Atlanta, GA 30322, USA

**Keywords:** rare disease, phenylketonuria, diet, choline, inborn errors of metabolism

## Abstract

Choline is an essential nutrient for brain development and function that is attained through high-protein foods, which are limited in the phenylalanine-restricted diet of people with phenylketonuria (PKU). This study compared choline consumption among individuals with PKU to a reference sample from the National Health and Nutrition Examination Survey (NHANES), and identified treatment and diet-related factors that may modulate choline needs. Participants were individuals with PKU (*n* = 120, 4–61 years) managed with dietary therapy alone (*n* = 49), sapropterin dihydrochloride for ≥1 year (*n* = 38), or pegvaliase for ≥1 year with no medical food (*n* = 33). NHANES participants were not pregnant or nursing and came from the 2015–2018 cycles (*n* = 10,681, 4–70 years). Dietary intake data were used to estimate total usual intake distributions for choline, and mean probability of adequacy (MPA) was calculated as a summary index of nutrient adequacy for four methyl-donor/co-factor nutrients that impact choline utilization (folate, vitamin B12, vitamin B6, and methionine). Only 10.8% (SE: 2.98) of the total PKU sample (14.7% [SE: 4.03] of children; 6.8% [SE: 2.89] of adults) achieved the adequate intake (AI) for choline, while 12.2% (SE:0.79) of the NHANES sample met the recommended level. Adults receiving pegvaliase were the most likely to exceed the AI for choline (14.82% [SE: 4.48]), while adults who were on diet therapy alone were the least likely (5.59% [SE: 2.95]). Without fortified medical foods, individuals on diet therapy and sapropterin would not be able to achieve the AI, and MPA for other methyl donor/co-factor nutrients would be reduced. More frequent monitoring of choline intake and increased choline fortification of medical foods could benefit this population.

## 1. Introduction

Phenylketonuria (PKU; OMIM #261600) is autosomal recessive disorder characterized by a deficiency of the phenylalanine hydroxylase (PAH; EC 1.14.16.1), an enzyme required to metabolize the amino acid phenylalanine (Phe) [1]. Untreated PKU leads to Phe accretion and subsequent significant changes in brain development and function [2]. PKU has traditionally been managed with a Phe-restricted diet combined with Phe-free or Phe-reduced amino acid (AA-MF) or glycomacropeptide medical foods (GMP-MF) to achieve protein adequacy while maintaining Phe within the recommended therapeutic range (120–360 µmol/L) [3,4]. Beyond providing essential amino acids, several medical foods are also fortified with synthetic vitamins, minerals, and essential fatty acids to provide a complete nutritional profile [5,6]. Nevertheless, nutrient inadequacies (e.g., docosahexaenoic acid, eicosapentanoic acid, cholesterol, potassium) still occur [7,8] and may be a particular concern among patients who have transitioned to liberalized or fully normalized diets with the use of new pharmacotherapies [9,10]. As suboptimal nutriture may contribute to cellular dysfunction, including the residual neurocognitive deficits observed in PKU [11,12], it is critical to further evaluate micronutrient intakes and status in this population.

**Figure 1:**
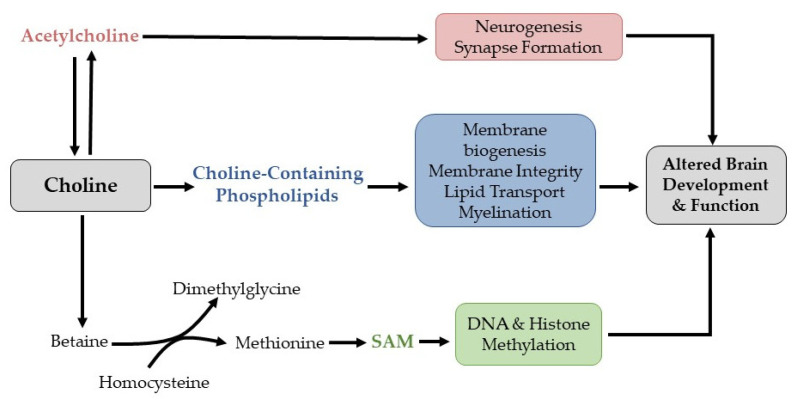
Choline serves as precursor for the following metabolites which impact brain development and function: (1) acetylcholine, a neurotransmitter that is involved in neurogenesis and synapse formation; (2) choline-containing phospholipids (e.g., phosphatidylcholine, sphingomyelin) which contribute to membrane biogenesis, lipid transport, and myelination; (3) betaine, which participates in one-carbon metabolism and aids in the regeneration of S-Adenosylmethionine (SAM). SAM is a methyl donor for both DNA and histone methylation.

Given the neurocognitive deficits observed in PKU, it is important to ensure adequate intakes of nutrients that play a critical role in neurotransmission and related processes. One such nutrient is choline, which serves as a precursor for phospholipids (e.g., phosphatidylcholine, sphingomyelin, ceramide) that are indispensable for the synthesis and structural integrity of myelin sheaths and cellular membranes throughout the brain and body [13] (Figure 1). In the brain, decreased concentrations of these phospholipids due to suboptimal choline intake may result in demyelination, white matter abnormalities, and mitochondrial dysfunction [14,15]. These structural and functional alterations may impair executive cognitive processes and enhance oxidative damage, both of which have been reported among individuals with PKU [16,17,18]. As choline can also be oxidized to the methyl-donor betaine, choline availability may additionally contribute to the differential epigenomic methylation patterns and subsequent changes in gene expression that have been observed in human [19] and rodent models [20] of PKU. Despite the important role of choline in neurocognitive development, there is a paucity of data on the effect of decreased choline consumption in patients with PKU.

Choline has limited endogenous synthesis [21] and is concentrated in high-protein foods [22], such as beef, chicken, fish and eggs, which are generally restricted in a PKU diet due to high Phe content. Thus, fortified medical foods are the predominant source of choline for individuals with PKU. The only study to have previously assessed choline intake among individuals with PKU [8] identified suboptimal choline intakes in 40% and 63% of participants, respectively, who regularly consumed GMP-MF and AA-MF. When considering the choline contribution solely from natural foods, no individuals in this cohort were capable of meeting the adequate intake (AI) level [8]. This is concerning given medical food compliance is typically low [23] and adjunct pharmacotherapies are now available that allow medical food consumption to be significantly reduced or discontinued. Beyond treatment regimen, choline nutriture may be further perturbed by suboptimal intake of other methyl-donor or co-factor nutrients that impact choline utilization, including the micronutrients folate, vitamin B12, vitamin B6, and the essential amino acid methionine. Poor intake of any of these nutrients may increase the dietary requirement for choline by increasing its utilization as a methyl donor and decreasing endogenous choline synthesis [24,25,26].

The objective of this study was to evaluate usual choline intake among individuals with PKU and the role of two factors that may modulate the dietary requirement for this nutrient: (1) treatment regimen, and (2) the concurrent intake of other nutrients that affect choline metabolism.

## 2. Materials and Methods

### 2.1. Study Participants

Dietary, biochemical, and demographic data for adults and children with PKU (4–61 years of age) were extracted from the research records of five clinical observational studies that were conducted at Emory University (Atlanta, GA, USA) and Boston Children’s Hospital (BCH; Boston, MA, USA). To assess the impact of treatment regimen on usual choline intake, participants were categorized into three groups based on the use of diet therapy only, the synthetic tetrahydrobiopterin therapy, sapropterin dihydrochloride (Kuvan^®^, BioMarin Pharmaceutical Inc., Novato, CA, USA), and the enzyme replacement therapy, pegvaliase (Palynziq^®^, BioMarin Pharmaceutical Inc., Novato, CA, USA). For inclusion in the sapropterin or pegvaliase groups, participants were required to have diet records that had been collected ≥1 year after treatment initiation. Medical food discontinuation was additionally required for inclusion in the pegvaliase group.

Data from the 2015–2018 National Health and Nutrition Examination Survey (NHANES) was used as a reference population. This cross-sectional survey is administered by the National Center for Health Statistics (NCHS) and uses a multistage probability cluster design to obtain a representative sample of the noninstitutionalized, civilian U.S. population [27]. To mirror the demographic characteristics of the PKU sample, the present study included NHANES participants 4–70 years of age who completed two nonconsecutive 24 h recalls. Individuals who were pregnant or nursing were excluded. Study protocols for both the PKU and NHANES cohorts were approved by the respective Research and Ethics Review Boards (Emory, BCH, NCHS), and written informed consent was obtained from all participants. The final analytic sample included 120 participants with PKU (*n* = 49 on diet therapy alone, *n* = 38 on sapropterin, *n* = 33 on pegvaliase) and 10,681 NHANES controls.

### 2.2. Quantification of Nutrient Intake from Food and Supplemental Sources

Participants in the PKU cohort completed diet records over two (*n* = 6) or three (*n* = 114) consecutive days, with detailed descriptions of all foods, beverages, supplements, and medical foods consumed. At the time of collection, records were reviewed for accuracy and completeness by a trained research registered dietitian. If participants had several complete diet records with at least two consecutive 24 h recalls, the record with the most recent collection date was selected for the present study. Records were then analyzed using the Nutrition Data System for Research (NDSR 2020, Nutrition Coordinating Center, University of Minnesota, Minneapolis, MN, USA) to determine dietary and supplementary intake of choline, vitamin B12, vitamin B6, folate, and methionine. While the NDSR Database contains over 18,000 foods [28], it does not include nutritional products that are critical for PKU treatment. This includes Phe-free or reduced Phe medical foods, which provide 85–90% of the protein needs for an individual with PKU, and low protein modified foods, which are common products made with low-protein flours [29]. Given the importance of these products, 19 medical foods were added to the database using the manufacturer-supplied nutrient information. For 33 low protein modified foods, recipes were created using ingredients within the NDSR database that contain composition data for choline and the other nutrients of interest. Any additional food items that were not found within the NDSR database were substituted for nutritionally comparable foods using the following set of nutrient tolerances per 100 g of food: 85 kcal, 2.5 g of fat, 100 mg of sodium, 10 g of carbohydrates, 5 g of protein, and 50 mg of phenylalanine [30].

For the NHANES cohort, diet and supplement data were obtained from two 24 h recalls collected using the automated multiple-pass method [31]. The first recall was collected in person at the mobile examination center, while the second recall was collected by telephone three to 10 days later. For each recall day, choline intake from food sources was determined based on nutrient values from the USDA’s Food and Nutrient database for Dietary Studies [32]. Supplemental choline consumption was derived from the dietary supplement questionnaire, which evaluates the use of vitamins, minerals, herbs, and other supplements over the 30 days preceding the dietary recall interview. Mean daily intake of supplemental choline was calculated based on the amount of choline taken across all supplements and the number of days each supplement was used.

### 2.3. Estimation of Usual Intake

Daily nutrient intake data was extrapolated from the 24-hour recalls and 3-day food records completed by NHANES and PKU participants, respectively. The use of two different diet data collection strategies was deemed appropriate based on a recent study [33], which found that the two strategies resulted in equivalent intake distributions for regularly consumed nutrients. Total usual nutrient intakes for choline, vitamin B12, vitamin B6, folate, and methionine, were estimated using the National Cancer Institute (NCI) method [34]. This approach uses Box-Cox transformed consumption-day data in a mixed effects model to adjust for relevant covariates and estimate within- and between-person variability. Using Monte-Carlo simulations, the estimates from this model were then used to derive usual intake distributions for populations and subgroups [35]. The present study estimated usual nutrient intake from both diet and supplemental sources using the “shrink-then-add” approach [36], and incorporated three covariates in the mixed model: sequence of the recall/record, day of the week that consumption data were collected, and subgroup (age and PKU treatment groups). For the PKU sample, nutrients from medical food were differentiated from dietary supplements (DS). The relative contributions of medical food and DS to total usual nutrient intakes were calculated at the population level by dividing usual supplement intake and medical food intake by the total usual nutrient intake.

### 2.4. Estimation of Nutrient Probability of Adequacy

For choline, nutrient adequacy and excess intake was assessed at the individual level using the cut-point method by comparing usual intakes to age- and gender-specific AI levels and tolerable upper intake levels (UL) [37]. The probability approach was used to estimate the probability of usual intake adequacy (PA) for folate, vitamin B12, vitamin B6, and methionine. Using the estimated average requirement (EAR) and standard deviation (SD) from the Institute of Medicine requirement distributions [38], PA was calculated as the percent of the requirement distribution that falls below an individual’s usual intake. Mean probability of adequacy (MPA) was calculated for each individual in both the PKU and NHANES groups as an average of the PA values for folate, vitamin B12, and vitamin B6. Mean probability of adequacy with methionine (MPAm) was also calculated for the PKU group, but not NHANES given dietary data for methionine was not available. Both MPA and MPAm were used as summary indices of nutrient adequacy for the four nutrients that impact choline utilization.

### 2.5. Statistical Analysis

All statistical analyses were completed in SAS (version 9.4, SAS Institute, Cary, NC, USA). The steps required for usual intake estimation were carried out using the SIMPLE Macro [39], which links three macros developed by the NCI. Standard errors for intake estimates were approximated by a simple bootstrap using 250 replicate weights for the PKU sample. For the NHANES samples, standard errors were estimated using Fay’s balanced repeated replication, and 32 replicate weights were generated with a factor of 0.3. Replicate weights for both samples were computed from the initial sampling weights. For NHANES, 4-year weights were constructed from the dietary two-day sample weights. For PKU, sample weights were calculated based on the demographic and treatment profile of the PKU population at the Emory Genetics Clinic, which this study assumed was representative of the United States PKU population.

To attain nutrient intake estimates that were representative of the United States population and could be compared to prior research [40], this study chose not to match PKU participants to a small sub-sample of participants from NHANES. Instead, the PKU and NHANES groups were evaluated separately using descriptive statistics to compare demographic characteristics and usual intakes by age (<18 years and ≥18 years) and treatment status (for the PKU sample). No inferential statistics were employed for group comparisons given different sampling strategies were used to generate the PKU and NHANES populations.

## 3. Results

Demographic and treatment characteristics of the PKU and NHANES samples are described in Table 1. Adults with PKU in all three treatment groups were predominantly female and younger on average compared to their unmatched NHANES counterparts. Children with PKU in the diet therapy group were also predominantly female, but were older than the NHANES sample. Overweight and obesity were more common among individuals with PKU in both age categories, with adults receiving pegvaliase (81.8%) and children receiving sapropterin (47.0%) demonstrating the highest prevalence. Within the PKU sample, there were notable differences in adherence across treatment groups, as denoted by plasma Phe concentrations. On average, children managed with both sapropterin and diet therapy had better adherence (lower Phe concentrations) and reported increased medical food consumption compared to adults on the corresponding treatments. Seventy-four individuals (*n* = 49 diet therapy, *n* = 25 sapropterin) within these treatment groups had medical food prescriptions and reported using 18 different medical foods (Table S1). Thirteen of these products contained choline and other nutrients that impact choline metabolism, however, the amount of choline was highly variable (range: 67 mg–530 mg/100 g medical food). Five medical foods were not fortified with any micronutrients or contained only select minerals and trace elements. Four adults (*n* = 2 diet therapy, *n* = 2 sapropterin) and two children (both sapropterin) who were prescribed “nutritionally complete” medical foods did not report medical food consumption during the recall period. Six adults on diet therapy and two adults receiving sapropterin were adherent with their formula prescription, but were solely consuming medical foods that lacked choline and other relevant nutrients.

### 3.1. Estimated Usual Choline Intake

Estimated mean usual total choline intakes for individuals 4–70 years of age in the PKU and NHANES populations were 230.81 (SE: 14.82) and 324.0 (SE: 2.84) mg/day, respectively. Usual intakes for individuals with PKU stratified by treatment groups are reported in Table 2 for adults and Table 3 for children. On average, adults in both the PKU and NHANES samples consumed more choline than their pediatric counterparts. Adults, however, were less likely to achieve the AI, compared to children. In the unaffected population, only 22% (SE: 1.08) of children (4–17 years) and 9.5% (SE: 0.92) of adults (18–70 years) exceeded the AI for choline. When accounting for all sources of choline (food, medical food, dietary supplements), adults with PKU on sapropterin or pegvaliase had a higher prevalence of choline adequacy than adults from NHANES. The opposite was observed among adults on diet therapy and all children with PKU. Across the full PKU sample, adults receiving pegvaliase were the most likely to exceed the AI for choline [14.82% (SE: 4.48)] while adults who were solely on diet therapy were the least likely [5.59% (SE: 2.95)]. Usual choline intake did not exceed the UL in any of the subgroups evaluated.

Among patients with PKU on diet and sapropterin therapies, medical food contributed 38% to 72% of total choline intake (Figure 1). Choline was not obtained from any other dietary supplements. Without the consumption of choline-fortified medical foods, individuals in these treatment groups would not be able to achieve the AI (Table 2 and Table 3).

### 3.2. Mean Probability of Adequacy (MPA) for Nutrients That Affect Choline Metabolism

**Figure 2:**
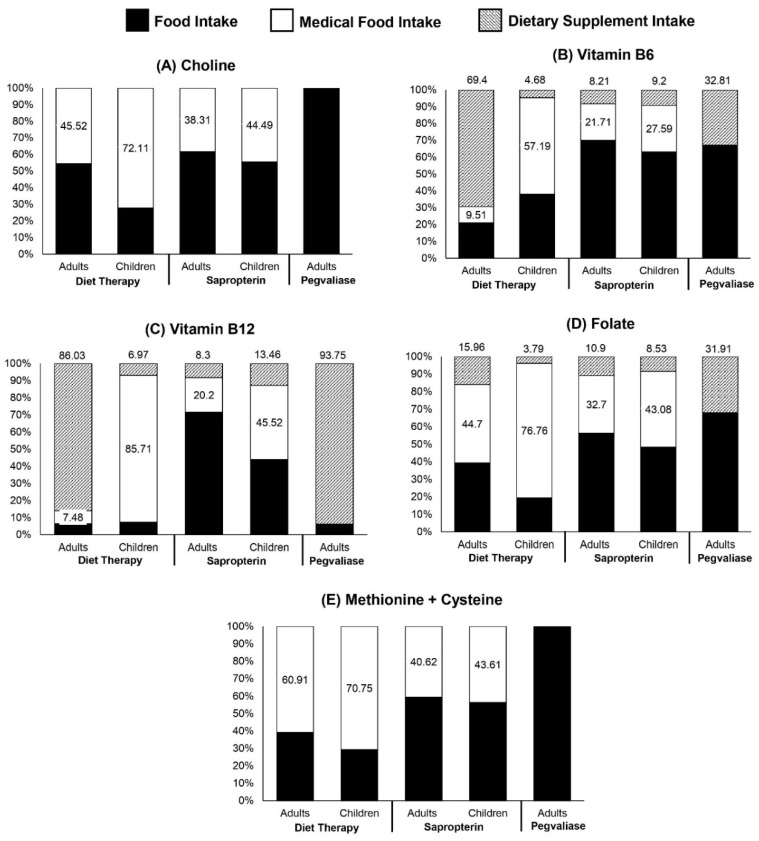
The contribution of food sources, medical food, and dietary supplements to total usual intakes for (**A**) choline, (**B**) vitamin B6, (**C**) vitamin B12, (**D**) folate, and (**E**) methionine plus cysteine among adults (*n* = 71) and children (*n* = 49) with PKU, stratified by treatment group (diet therapy, sapropterin, and pegvaliase). Percentage values within white bars represent the contribution from medical food and percentage values above each bar represent the contribution of dietary supplements.

When considering total intake from food sources, medical food, and dietary supplements, median MPA of the methyl-donor nutrients that impact choline utilization was 100% among adults and children in the PKU and NHANES cohorts, suggesting that there was a very low probability of inadequate intake (Table 4). While dietary supplements contributed 40%, on average, to intakes of these micronutrients and amino acids among the PKU pegvaliase group (Figure 1), median MPA and MPAm did not exhibit significant change when solely considering intake from food sources. The NHANES cohort exhibited the same trend. Among the diet therapy and sapropterin groups, however, the exclusion of medical foods and dietary supplements resulted in a notable decrease in median MPA and MPAm. This was particularly evident among children in the diet therapy group, who obtained 57% to 85% of their methyl-donor nutrient intake from medical foods (Figure 2).

## 4. Discussion

The importance of dietary choline for neurological development and sustained cognitive function has been supported by animal models and human studies [41,42,43]. Yet, data on the adequacy of dietary intakes of choline remain limited, especially among populations with an increased risk of choline deficiency and neurocognitive deficits, such as PKU. This descriptive study is the first to estimate usual choline intake among adults and children with PKU across three therapies that could modulate choline needs. Our findings suggest that the overall prevalence of choline adequacy was lower in the PKU sample than the US population from NHANES; however, the proportion of individuals able to exceed the AI for choline was relatively low in both populations. This aligns with previous assessments of choline intake in NHANES [22,40], and suggests that educational efforts and supplement consumption remain limited and/or are not substantially impacting choline intake. Our observations also complement one prior study in PKU [8], which also found that individuals on sapropterin and/or dietary treatment were not able to achieve the AI for choline without the consumption of fortified medical foods. This study advances the previous research by using the NCI method to estimate usual choline intake distributions and separately characterize consumption patterns by treatment group. These treatment-specific estimates lend insight into the impact of diet liberalization and normalization on nutrient adequacy.

Across PKU treatment groups, adults on diet therapy were the least likely to achieve choline intakes above the AI. There are several factors that may have contributed to this finding. The first is poor treatment adherence, which was evident in the group’s elevated median Phe concentration, and could have derived from the inconsistent intake of fortified medical foods and/or the overconsumption of food items lacking choline. The low prevalence of choline adequacy also may have been affected by the intake patterns of six patients (35% of the group) who exhibited good treatment adherence but were not consuming choline-fortified medical foods or multivitamin/mineral supplements. Alternately, some patients in this group were consuming fortified medical foods as prescribed, but the amount of choline in these products may not have been sufficient to compensate for the lack of choline in their protein-restricted diets. Given treatment nonadherence has been found to increase with age in PKU [23], and may impact choline status, it would be beneficial to start monitoring choline intake more regularly in this group. Moreover, medical food formulations may benefit from higher amounts of added choline given most mainstream multivitamin/multimineral supplements still do not contain choline [44], and thus cannot serve as an alternate nutrient source for this population.

Adults receiving pegvaliase, who were consuming an unrestricted diet, were found to have the highest prevalence of choline intake above the AI. The proportion of patients meeting this recommendation was slightly higher than NHANES, suggesting that patients managed with pegvaliase require less supplemental choline than their counterparts on sapropterin or diet therapy to match the intake levels of their unaffected peers. Both groups also had similar usual intakes for the methyl-donor and co-factor nutrients that impact choline metabolism. Median MPA and MPAm were 100% among adults on pegvaliase and NHANES controls, and both indices did not notably change when excluding the nutrient input from dietary supplements. Micronutrient and protein adequacy for 18 of the 33 individuals in the pegvaliase group had been previously evaluated in another study [45], which found that nutrient intakes for most patients met or exceeded the dietary reference intake (DRI), and that overall diet quality (assessed by the Healthy Eating Index) was nearly equivalent to the 2015–2016 NHANES sample. The present study expands these findings by identifying similar patterns for a group of essential nutrients that were previously not assessed.

Although the pegvaliase group was able to consume adequate amounts of the methyl donor and co-factor nutrients solely through diet, the other PKU therapy groups could only attain equivalent MPA and MPAm scores when medical food and dietary supplements were consumed. Without these supplements, patients on diet therapy and sapropterin may not have the required methyl donor concentrations to compensate for suboptimal choline intakes. This is a concern given the present study identified a low prevalence of choline adequacy even with the consumption of fortified medical foods. Poor metabolic control could further increase the risk of choline deficiency given high Phe levels may limit the endogenous synthesis of choline. This could occur through the increased production of phenylacetate, a phenylketone that has been reported to inhibit the estrogenic induction of the rate-liming enzyme, phosphatidylethanolamine-*N*-methyltransferase [46,47]. While future research is required to directly assess how choline synthesis is modulated by Phe control, the present findings emphasize the importance of patient education for maintaining treatment adherence in PKU, and subsequently increasing choline consumption and the intake of nutrients that support choline metabolism

As choline status may be an important contributor to differences in neurocognitive outcomes among individuals with PKU [12], this analysis provides foundational insight into the dietary and pharmacological factors that impact choline adequacy in this population. A limitation of this study is that dietary intakes were not determined in tandem with biochemical measures. The utility of this, however, remains unclear as there are important limitations associated with choline biomarkers [48]. For example, plasma choline has not been found to reflect the diet at very low intake levels [47,49] and plasma betaine concentrations, which may be a better marker of dietary intake, does not accurately represent tissue concentrations [50]. Through the use of metabolomics and isotope dilution, emerging studies can better estimate the relationship between choline intake and choline status to provide new clinically useful biomarkers [48]. Other shortcomings of the present study were potential confounding and measurement error. This study’s PKU sample was predominantly Caucasian, which reflects the racial distribution of the PKU population in the US [51], but may have weakened our comparison to the NHANES reference, which was not restricted by race. The use of self-reported dietary and supplement data may have also underestimed or overestimated both micronutrient and amino acid intakes. This may have been further impacted by the collection of dietary supplements on the day of each diet record, which may not be as representative of long-term intake as the 30-day questionnaire administered by NHANES. The impact of our assessment technique, however, may be minimal given choline is not included in most dietary supplements and there remains limited knowledge regarding best practices for supplement reporting [36].

Despite these limitations, the present analysis was strengthened by the application of statistical models that adjust dietary intakes for the effects of random measurement error, which allowed for the estimation of usual intake distributions. An additional strength was the comparison of PKU intake distributions to a nationally representative sample of unaffected individuals. This provided prospective on the normalization of nutrient intake based on PKU therapy and age group. Although this study’s sample was relatively large given PKU is a rare disease, future analyses would benefit from additional participants so that differences in intake trends by sex could also be assessed.

## 5. Conclusions

In summary, this study demonstrated that adults and children with PKU who are managed on phenylalanine-restricted and liberalized diets were unable to achieve the AI for choline without the consumption of medical food. Future research is needed to determine if suboptimal choline intake affects cognitive function, particularly among individuals on diet therapy and sapropterin with poor treatment adherence.

## Figures and Tables

**Table 1:** Characteristics of the NHANES (*n* = 10,681) and PKU (*n* = 120) Study Populations, by Age and Treatment Group.

	PKU			NHANES
	Diet Therapy	Sapropterin Dihydrochloride	Pegvaliase	
**Adults** **(≥18 years)**	*n* = 17	*n* = 21	*n* = 33	*n* = 7267
Age, years ^2^	21	27	36	43
	(18, 26)	(22, 34)	(32, 44)	(30, 56)
Age range, years	18–50	18–45	22–61	18–70
Female, *n* (%) ^1^	12 (70.6)	13 (61.9)	20 (60.6)	3589 (50.6)
Caucasian, *n* (%) ^1^	16 (94.1)	21 (100.0)	33 (100.0)	2191 (61.3)
Weight status, *n* (%) ^1^				
Overweight	7 (41.2)	5 (23.8)	11 (33.3)	2040 (30.0)
Obese	4 (23.5)	8 (38.1)	16 (48.5)	2939 (41.5)
Plasma phe (µmol/L) ^2^	842.0	362.0	23.0	--------
	(613.2, 951.1)	(261.0, 533.2)	(3.0, 218.0)	
Taking medical food, *n* (%)	17 (100.0)	12 (57.1)	0 (0.0)	--------
**Children** **(<18 years)**	*n* = 32	*n* = 17	*n* = 0	*n* = 3414
Age, years ^2^	14	10	--------	10
	(10, 16)	(8, 13)		(7, 14)
Age range, years	4–17	6–16	--------	4–17
Female, *n* (%) ^1^	23 (71.9)	8 (47.1)	--------	1666 (49.1)
Caucasian, *n* (%) ^1^	31 (96.9)	16 (94.1)	--------	999 (49.6)
Weight status, *n* (%) ^1^			--------	
Overweight	4 (12.5)	4 (23.5)	--------	526 (15.8)
Obese	4 (12.5)	4 (23.5)	--------	719 (21.2)
Plasma phe (µmol/L) ^2^	626.0	302.0	--------	--------
	(323.5, 1082.0)	(185.0, 397.0)		
Taking medical food, *n* (%)	32 (100.0)	13 (76.5)	--------	--------

^1^ Categorical variables are reported as the unweighted frequency and weighted percent. ^2^ Continuous variables are reported as the median (IQR).

**Table 2:** Total Usual Intakes and the Estimated Percent (%) of Usual Intakes Above the Adequate Intake (AI) for Choline Among Adults 18–70 Years of Age with PKU (*n* = 71) and Adults from NHANES 2015–2018 (*n* = 7267).

			Percentile			
Subgroup	*n* ^1^	Mean (SE)	25th (SE)	50th (SE)	75th (SE)	% >AI
NHANES	7267	341.3 (3.6)	257.9 (3.6)	327.5 (3.5)	409.0 (4.7)	9.5
PKU diet therapy	17					
With MF ^2^		203.6 (34.5)	116.4 (18.0)	175.7 (24.0)	263.9 (32.5)	5.6
Without MF ^2^		115.3 (13.3)	73.3 (11.0)	101.5 (13.2)	145.6 (17.2)	0.2
PKU sapropterin	21					
With MF ^2^		299.4 (37.0)	179.2 (22.2)	268.3 (27.3)	390.6 (37.4)	14.2
Without MF ^2^		176.6 (21.4)	114.2 (17.6)	160.4 (20.7)	222.1 (26.7)	0.8
PKU pegvaliase	33	302.3 (28.0)	185.1 (21.8)	273.4 (26.2)	389.5 (35.3)	14.8

^1^ Represents the unweighted sample size. ^2^ Represents the usual intakes and the estimated percent (%) of usual intakes above the AI for the designated treatment group under two conditions: (1) with reported medical food intake, (2) without medical food (hypothetical). Abbreviations: AI, adequate intake; MF, medical food; PKU, phenylketonuria.

**Table 3:** Total Usual Intakes and the Estimated Percent (%) of Usual Intakes Above the Adequate Intake (AI) for Choline Among Children 4–17 Years of Age with PKU (*n* = 49) and Children from NHANES 2015–2018 (*n* = 3414).

			Percentile			
Subgroup	*n* ^1^	Mean (SE)	25 (SE)	50 (SE)	75 (SE)	% >AI
NHANES	3414	261.2 (2.8)	191.5 (2.9)	249.3 (2.9)	318.2 (3.6)	22.0
PKU diet therapy	32					
With MF ^2^		221.0 (16.0)	148.2 (12.7)	211.2 (15.0)	287.0 (24.2)	12.3
Without MF ^2^		61.6 (5.93)	45.4 (6.0)	58.9 (5.9)	75.6 (7.4)	0
PKU sapropterin	17					
With MF ^2^		174.8 (15.1)	105.3 (15.2)	167.4 (15.8)	232.4 (22.4)	6.4
Without MF ^2^		96.7 (8.8)	75.6 (9.6)	94.6 (8.9)	115.1 (9.9)	0

^1^ Represents the unweighted sample size. ^2^ Represents the usual intakes and the estimated percent (%) of usual intakes above the AI for the designated treatment group under two conditions: (1) with reported medical food intake, (2) without medical food (hypothetical). Abbreviations: AI, adequate intake; MF, medical food; PKU, phenylketonuria.

**Table 4:** Mean Probability of Adequacy (MPA) of nutrients that affect choline metabolism (vitamin B6, vitamin B12, folate, methionine) among individuals with PKU (*n* = 120) and NHANES participants (*n* = 10,681), by age and treatment group ^1^.

	Adults			Children		
	*n* ^2^	MPA^3^	MPAm ^4^	*n* ^2^	MPA ^3^	MPAm ^4^
**NHANES**	7267			3414		
With DS ^5^		100	-----		100	-----
		(100, 100)			(100, 100)	
Without DS ^5^		100	-----		100	-----
		(100, 100)			(100, 100)	
**PKU diet therapy**	17			32		
With MF + DS ^5^		100	100		100 (100, 100)	100
		(100, 100)	(100, 100)			(100, 100)
Without MF + DS ^5^		65.1	48.8		37.2	28.1
		(26.7, 96.3)	(25.2, 72.6)		(1.6, 64.8)	(3.2, 51.8)
**PKU sapropterin**	21			17		
With MF + DS ^5^		100	100		100	98.9
		(95.7, 100)	(83.9, 100)		(94.7, 100)	(88.1, 100)
Without MF + DS ^5^		77.4	74.4		66.7	73.3
		(66.7, 100)	(52.6, 98.5)		(31.9, 97.3)	(25.4, 97.3)
**PKU pegvaliase**	33			0		
With DS ^5^		100	100		-----	-----
		(96.1, 100)	(97.1, 100)			
Without DS ^5^		99.8	99.8		-----	-----
		(80.0, 100)	(85.0, 100)			

^1^ Reported as the median (IQR) of the distribution of MPA. ^2^ Represents the unweighted sample size. ^3^ Probabilities of adequacy for vitamin B6, vitamin B12, and folate were averaged for each participant to estimate MPA. ^4^ MPAm was estimated in the same manner as MPA but includes the probability of adequacy for the sum of methionine and cysteine. Dietary data for methionine was not available for NHANES. ^5^ Represents the MPA and MPAm (PKU only) for the designated diagnosis and/or treatment group under under two conditions: (1) with reported medical food and dietary supplement intake, (2) without medical food or dietary supplements (hypothetical).Abbreviations: DS, dietary supplements; MF, medical food; MPA, mean probability of adequacy; MPAm, mean probability of adequacy with methionine; PKU, phenylketonuria.

## Data Availability

The data presented in this study are available on request from the corresponding author.

## Supplementary Materials

The following supporting information can be downloaded at: https://www.mdpi.com/article/10.3390/nu14194056/s1, Table S1: Amount of Choline, Vitamin B12, Vitamin B6, Folic Acid, and Methionine found in 100 g or 100 mL of the Medical Foods Reported by Participants with PKU.
