# Supplementary material for: Characterization of Choline Nutriture among Adults and Children with Phenylketonuria
Source: Nutrients. 2022 Sep 29;14(19):4056. doi: 10.3390/nu14194056 (PMC9572308; doi:10.3390/nu14194056)
Supplement: Supplementary file 1 [file nutrients-14-04056-s001.zip › nutrients-1909033-supplementary.pdf]

Table S1. Amount of Choline, Vitamin B12, Vitamin B6, Folic Acid, and Methionine found in 100g or 100mL of the Medical Foods Reported by Participants with PKU

Medical Food	N[1]	Choline (mg)	Vitamin B12 (mcg)	Vitamin B6 (mg)	Folic Acid (mcg)	Methionine (g)
Phenex-2	28	100	5	1.10	425	0.60
Phenylade MTE Amino Acid Blend	10	0	0	0	0	1.89
Phenyl-free 2HP	5	67	3.10	1.29	470	0.88
Phenyl-free 2	4	98	2.40	0.98	350	0.48
Phlexy-10 Drink Mix	4	0	0	0	0	0.85
Phenylade Amino Acid Blend	3	0	0	0	0	1.95
Phenylade 60	3	425	4.20	2.30	700	1.45
Glytactin RTD 15	3	82.4	0.30	0.20	56	0.06
Glytactin Bettermilk	3	375	1.10	1.50	200	0.45
PKU Periflex Advance	2	376	2.70	1.40	430	0.64
Phenylade Essential	2	210	3	1.40	300	0.61
Phenylade GMP Mix-In	2	0	0	0	0	1.17
PKU Cooler	2	102	0.82	0.44	51	0.26
PKU Maxamum	1	430	3.10	1.60	491	0.87
PKU Express	1	530	4.30	2.50	360	1.24
Phenylade 40	1	284	2.80	1.50	468	1.08
PKU Easy Microtabs	1	0	0	0	0	1.42
Camino Pro PKU	1	95.5	0.90	0.30	81.4	0.29

[1]Reflects the number of participants who reported taking a specific medical food. Participants who were consuming two or more medical foods were reported more than once.
